# Case Report: Lorlatinib for the treatment of *ALK*-rearranged poorly differentiated thyroid carcinoma after progression to prior ALK-specific tyrosine-kinase inhibitor

**DOI:** 10.3389/fonc.2026.1802225

**Published:** 2026-03-09

**Authors:** Jesús Yaringaño, Jorge Hernando, Alejandro García-Álvarez, Carmela Iglesias-Felip, Carles Zafón, Ana Vivancos, María Roca-Herrera, Sergio Pérez-Fernández, Sandra Martínez-Badal, Belén Elguero, Jaume Capdevila

**Affiliations:** 1 Medical Oncology Department, Vall d’Hebron Barcelona Hospital Campus, Barcelona, Spain; 2 Department of Medical Oncology, Gastrointestinal and Endocrine Tumor Unit, Vall d’Hebron Hospital Universitari, Vall d’Hebron Barcelona Hospital Campus, Barcelona, Spain; 3 Neuroendocrine and Endocrine Tumor Translational Research Program (NET-VHIO), Vall Hebron Institute of Oncology (VHIO), Vall d’Hebron Barcelona Hospital Campus, Barcelona, Spain; 4 Pathology Department, Vall d’Hebron Barcelona Hospital Campus, Barcelona, Spain; 5 Endocrinology Department, Vall d’Hebron Barcelona Hospital Campus, Barcelona, Spain; 6 Cancer Genomics Group, Vall d’Hebron Institute of Oncology, Barcelona, Spain; 7 Oncology Data Science Group (ODysSey), Vall d’Hebron Institute of Oncology, Barcelona, Spain; 8 Stem Cell and Cancer Group, Vall d’Hebron Institute of Oncology, Barcelona, Spain

**Keywords:** ALK (anaplastic lymphoma kinase), lorlatinib, sequential treatment ALK-TKI, targeted therapy, thyroid cacinoma

## Abstract

ALK rearrangements are rare but actionable oncogenic drivers in thyroid cancer, particularly in aggressive histologies such as poorly differentiated thyroid carcinoma (PDTC), and evidence supporting sequential ALK inhibition in this setting is scarce. We report a 19-year-old male with ALK-rearranged, radioiodine-refractory PDTC who started systemic therapy with ceritinib, achieving a complete metabolic response. After treatment discontinuation and subsequent progression despite ceritinib reintroduction, lorlatinib was initiated. Treatment with the third-generation ALK inhibitor led to a deep and durable complete metabolic response, sustained for more than four years, including persistence of remission after treatment discontinuation, with minimal toxicity. This case highlights the potential role of sequential ALK inhibition to overcome acquired resistance in ALK-rearranged TC and underscores the importance of comprehensive molecular profiling to guide personalized treatment strategies in rare aggressive thyroid cancers.

## Introduction

Thyroid cancer (TC) is the most common endocrine malignancy, accounting for approximately 3% of global cancer incidence (1). While most subtypes, such as papillary thyroid carcinoma (PTC), have an excellent prognosis with the standard approach including surgery followed by radioactive iodine (RAI), poorly differentiated thyroid carcinoma (PDTC) represents a more aggressive entity with significantly worse outcomes (2, 3).

In the latest WHO classification, PDTC is defined as a high-grade, follicular-derived carcinoma characterized by solid/trabecular/insular growth and elevated mitotic activity (4). Molecular profiling in PDTC has revealed recurrent alterations in *KRAS* (45%), *TP53* (25%), *PTEN* (16%), *BRAF* (10%), and less frequently, gene fusions involving *RET* and *ALK*.

*ALK* rearrangement is detected in different cancer types with a prevalence of up to 5% in Non-Small-Cell-Lung-Cancer (NSCLC) (5). *ALK* translocation has been described in thyroid cancer since 2014 (6, 7), with a higher frequency, up to 50%, in patients with radiation exposure (8). The Cancer Genome Atlas Research Network evaluated a large cohort of PTCs in which the incidence of *ALK* fusions was about 0.8% (9), with a higher incidence in PDTC (≈2.2%) (10). Also, the spectrum and outcomes of *ALK* fusions found in thyroid cancer are not fully characterized. Up to this date, the largest case series consists of 44 patients with ALK positive TC with the most common fusion partners of ALK being STRN (50%) and EML4 (39%), although other partners, such as TFG, GTF2IRD1, and CCDC149, have also been described (11, 12).

Moreover, targeted therapy with ALK multikinase inhibitors (MKI) including crizotinib, ceritinib, and brigatinib, has demonstrated efficacy in ALK-rearranged non–small-cell lung cancer and increasingly in other tumor types, such as TC (13). Clinical experience in TC remains limited to case reports and small series; however, responses to first- and second-generation ALK inhibitors have been described (**Table 1**), and more recently, lorlatinib has shown clinical activity in metastatic PTC harboring an EML4–ALK fusion (14).

**Table 1:** Summary of published ALK positive thyroid cancer cases treated with TKI.

Author	Age	Sex	Diagnosis	Dissemination pattern before TKI	Fusion partner	TKI	Dose	Toxicity	Grade	BOR	Time to BOR	PFS
Zhu L, et al	64	F	PTC	Brain and Lung metastases	STRN	Alectinib	600mg BID	Diarrhea	G1	PR	3 months	8m**
Buriolla S, et al	69	F	PTC	Lung metastases	STRN	Alectinib	600mg BID	CPK elevation	G3	CR	11 months	17m**
De Salins V, et al.	76	F	PTC	Bone and Breast metastases	Not mentioned	Crizotinib	250mg BID	Constipation	G2	CR	4 months	7m
Godbert Y, et al*	71	F	PTC + ATC	Lung metastases	Not mentioned	Crizotinib	250mg QD	Not described	Not described	PR	3 months	36m
Leroy L, et al*	75	F	ATC	Lung metastases	STRN	Ceritinib	750mg QD	Not described	Not described	PR	2 months	16m
		F	ATC	Brain metastases	STRN	Brigatinib	180mg QD	Not described	Not described	PR	2 months	8m***
Aydemirli et al	60	M	PTC	Lymph Nodes and Lung metastases	EML4	Crizotinib	250mg BID	Not described	Not described	SD	2 months	8m
				Lymph Nodes, Lung and Brain metastases	EML4	Lorlatinib	100mg QD	Dyslipidemia	G2	PR	3 months	7m**
Yaringaño J, et al	19	M	PDTC	Lymph Nodes	TFG	Ceritinib	750mg QD	Abdominal pain	G2	CR	16 months	45m
				Lymph Nodes	TFG	Lorlatinib	100mg QD	Nausea	G1	CR	27 months	55m**

*Leroy L, et al. published an update on the case reported by Godbert Y, et al.

**Treatment was ongoing at the time of the publication.

*** Patients cause of death was related to a second neoplasm.

We report a case of *ALK*-rearranged PDTC with clinical progression on ceritinib that achieved a complete response to lorlatinib, highlighting the potential role of sequential ALK inhibition in aggressive thyroid malignancies with actionable molecular alterations.

## Case description

A 19-year-old male, without relevant pathological background, presented in December 2016 with a growing, painful right cervical mass. Ultrasound revealed a 4.7 x 3.4 x 2.3 cm nodule in the right thyroid lobe and a 3.1 x 1.6 cm right cervical lymph node with central necrosis. Fine-needle aspiration (FNA) suggested carcinoma, with negative calcitonin. Thyroid function was normal, and no compressive symptoms were reported.

In January 2017, the patient underwent total thyroidectomy with bilateral central neck dissection and right functional lateral neck dissection (levels II-V). Despite tumor adherence to adjacent structures, complete macroscopic resection was achieved. Histopathology confirmed poorly differentiated thyroid carcinoma, staged pT3N1b. Immunohistochemistry (IHC) showed weak thyroglobulin and PAX8 positivity (MRQ-50, MMAb Cell Marque™ Corp), strong TTF-1 staining (8G7G3/1, MMAb Ventana^®^), and Ki67 of 28% (30-9, RbMAb Ventana^®^). Calcitonin (SP17, RbMAb Cell Marque™ Corp) and p53 (DO-7, MMAb Ventana^®^) were negative. ALK IHC (Ventana^®^ ALK D5F3 CDx Assay) was strongly positive. Molecular testing was negative for *BRAF* (PCR, kit Cobas^®^ *BRAF*/*NRAS* FFPET Mutation Test) or other actionable molecular alterations.

**Figure 1:**
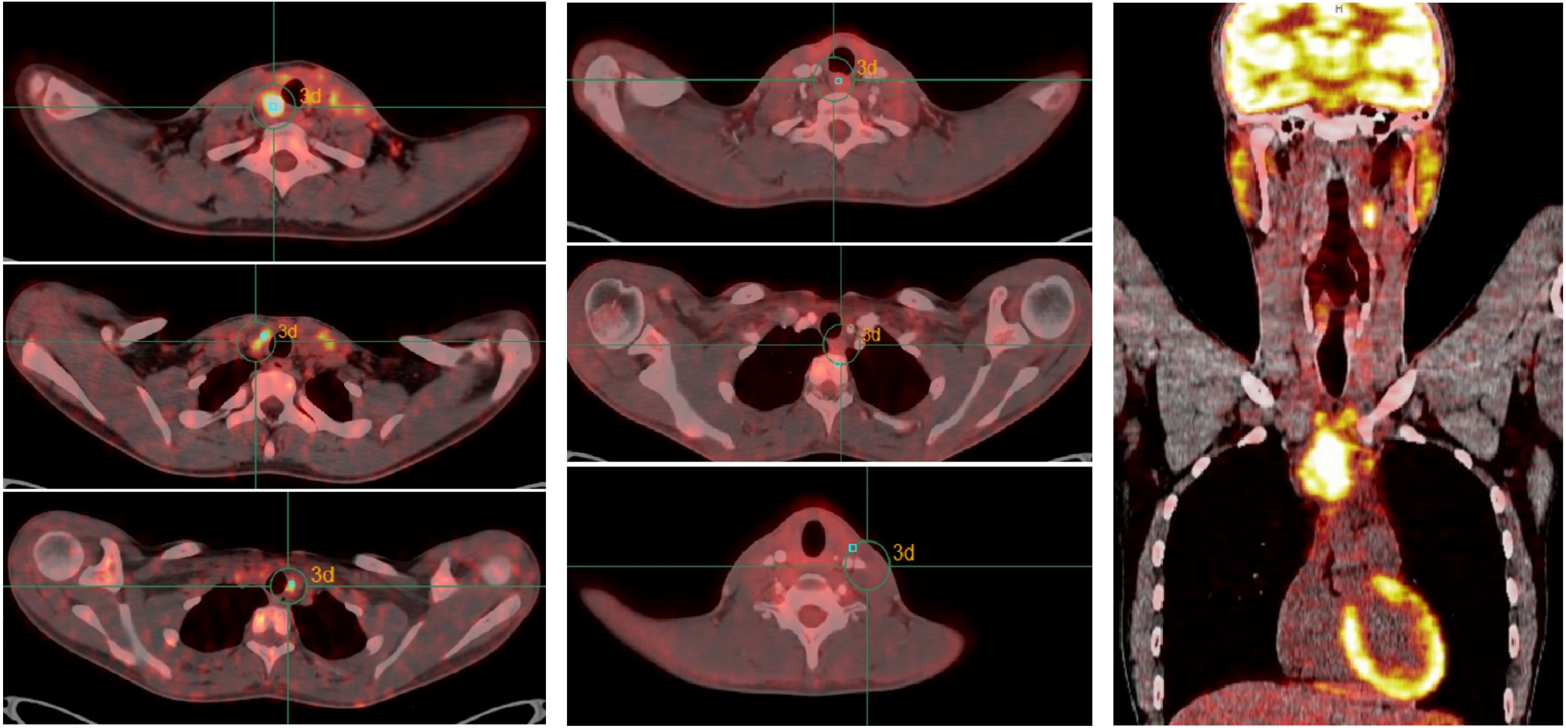
Radiological evolution under first-line treatment with ceritinib. (Left) Before treatment started, PET/CT scan shows signs of bilateral laterocervical adenopathy, as well as central compartment, and left supraclavicular lymph nodes. (Middle) Alter six cycles, normalization or very marked improvement of metabolic activity is observed in all previously detected laterocervical, supraclavicular and paratracheal lymph nodes. (Right) At progression, PET-CT scan showing evidence of local recurrence at the level of the left surgical bed and extensive infiltrative adenopathic involvement in the anterior mediastinum, as well as at level II left laterocervical and homolateral supraclavicular. No evidence of lesions suggestive of pulmonary or bone metastases.

Immediate postoperative cervical-thoracic scintigraphy revealed no uptake suggestive of significant residual thyroid tissue. No hypermetabolic lateral cervical lymph nodes or distant deposits were identified. However, ultrasound revealed clearly pathological lymphadenopathies in the left lateral cervical region. FNA confirmed nodal disease. In March, additional central and left lateral neck dissection was performed. The patient received I-131 therapy, and post-treatment scans showed only uptake in the thyroid bed, consistent with residual thyroid tissue, and no distant disease. PET-CT in April 2017 revealed metabolically active lymphadenopathy in the neck and supraclavicular regions (**Figure 1** left).

Given recurrent nodal disease, the case was discussed at a multidisciplinary tumor board, and the patient was referred for systemic therapy. Pathology review confirmed ALK rearrangement by IHC (Ventana^®^ ALK D5F3 CDx Assay) and mismatch repair proficiency (MSH2 G219-1129, MLH1 G168–728 and MSH6/GTBP 44, BD PharMingen Bioscience). In June 2017, he initiated ceritinib 750mg daily in a clinical trial. A partial response was observed after two cycles with MRI. Treatment was temporarily interrupted at cycle four due to grade 3 abdominal pain and resumed at a reduced dose of 600mg daily and improvement of toxicity to G1-2. After six cycles, PET-CT demonstrated marked metabolic improvement of all previously involved lateral cervical, supraclavicular, and paratracheal lymph nodes (**Figure 1** middle).

**Figure 2:**
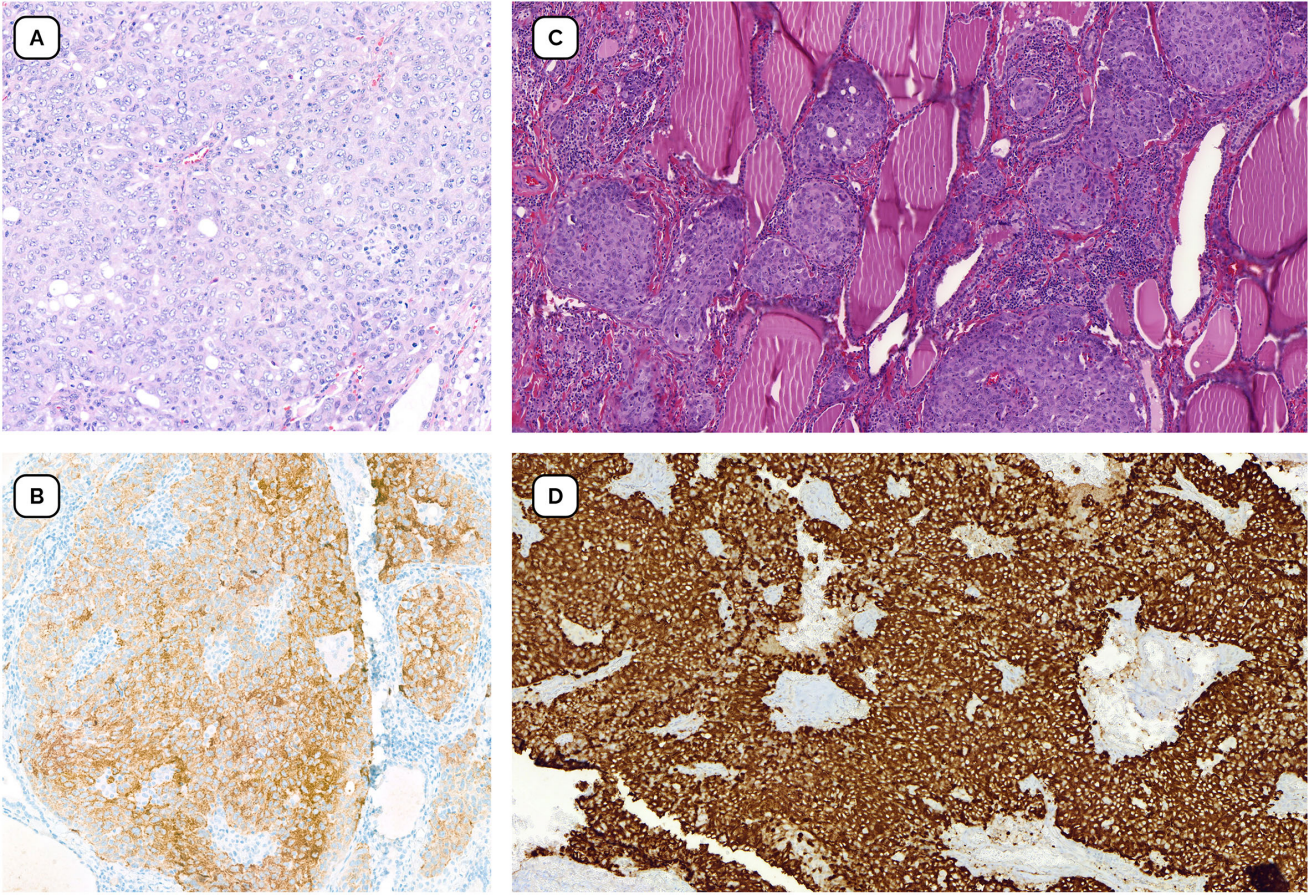
Pathology findings. **(A)** Thyroidectomy specimen with diagnosis of poorly differentiated thyroid carcinoma (H&E, x20) **(B)** Immunohistochemical staining positive for ALK (Ventana anti-ALK DF53, x20) performed in surgical specimen. **(C)** Infiltrative tumor nests with a solid pattern adjacent to isolated follicular structures, consistent with poorly differentiated carcinoma from biopsy at recurrence (H&E, x20). **(D)** Immunohistochemical staining intensely positive for ALK (Ventana anti-ALK DF53, x20).

In November 2018, imaging showed a complete metabolic response. Treatment was discontinued at the patient’s request after sustained response and considering the past toxicity. Three weeks later, recurrence in the left cervical region was detected by the patient and confirmed by MRI and ultrasound. Salvage lymphadenectomy confirmed persistent ALK-positive disease. Ceritinib was restarted off trial with sustained disease control until March 2021. At that time, MRI and PET-CT revealed extensive recurrence in the cervical and mediastinal lymph nodes (**Figure 1** right). Biopsy confirmed PDTC recurrence with strong ALK expression (**Figure 2**). RNA-based next-generation sequencing (Oncomine Precision Assay, ThermoFisher Scientific; reference genome GRCh37/hg19) performed on the 2021 biopsy identified a TFG(NM_006070.6)::ALK(NM_004304.5) fusion (T4:A19).

In May 2021, the patient started lorlatinib 100mg daily. After four cycles, a partial response was observed. SPECT-CT performed after cycle six showed no I-123 uptake, ruling out redifferentiation and radioiodine resensitization. Treatment was well tolerated, with only grade 1 nausea present and no other relevant toxicity.

**Figure 3:**
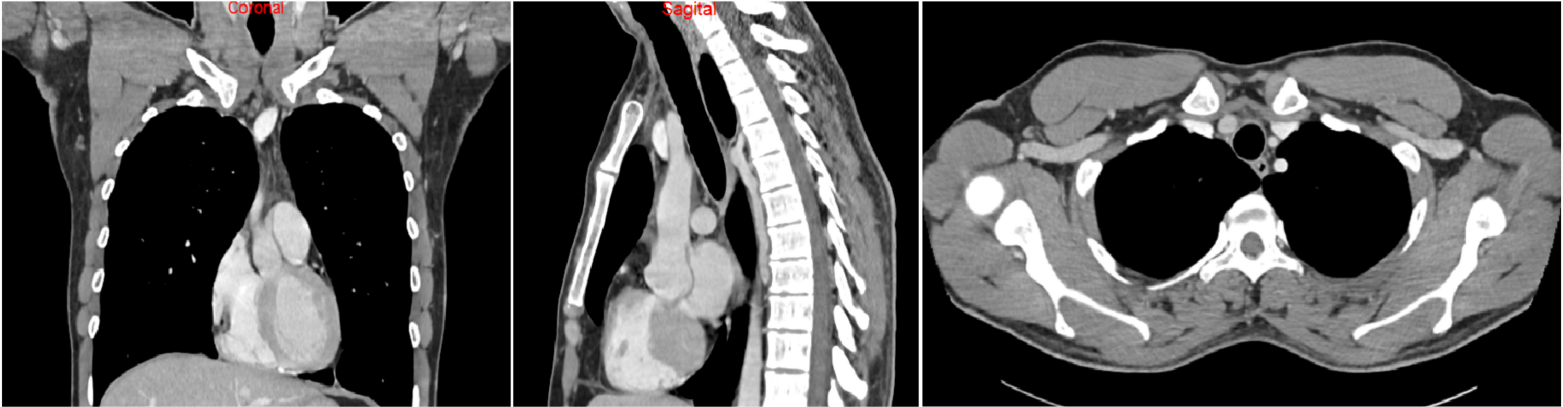
CT scan demonstrates complete response under of local and lymph node recurrence treatment with lorlatinib.

**Figure 4:**
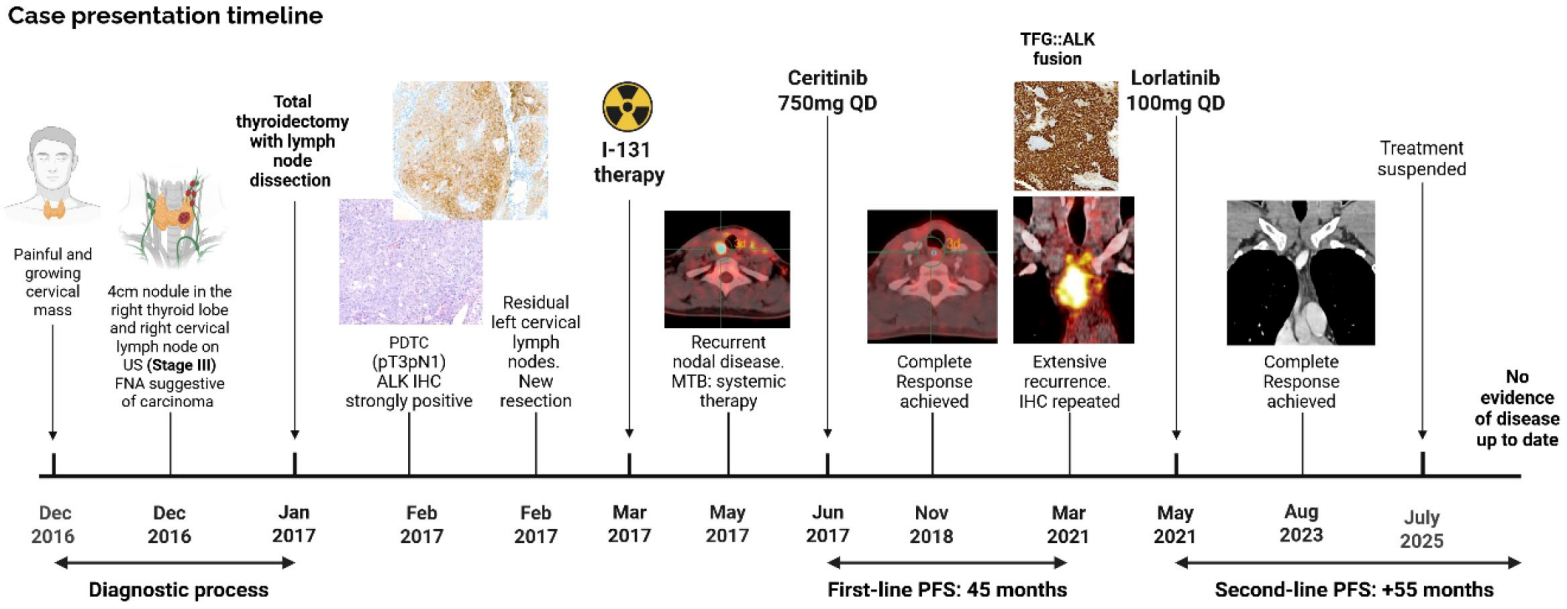
Case timeline.

Serial imaging from August 2023 onward showed no evidence of disease. In December 2023, PET-CT confirmed a metabolic complete response. Liquid biopsy (Guardant360^®^) in August 2024 identified no pathogenic variants. Lorlatinib was discontinued in July 2025 due to depressive symptoms, a toxicity already described with this drug. At last follow-up in October 2025, the patient remained in complete remission without treatment (**Figure 3**). Written informed consent was obtained from the patient for publication of this case report (**Figure 4**).

## Discussion

As the incidence of thyroid cancer increases, there have been active studies on the genomic profiling of patients with thyroid cancer to develop more effective treatment methods. Differentiated forms of thyroid carcinoma may present oncogenic somatic alterations in the MAPK signaling pathway, including point mutations of *BRAF* and *RAS* genes as well as fusions involving RET and NTRK1 tyrosine kinases (12). Meanwhile, PDTC adds to RAS-RAF-MEK-ERK/JNK pathway alterations, the affectation of PI3K/PTEN-AKT-mTOR signaling pathway, and cell cycle related genes (15).

As mentioned before, *ALK* rearrangements are rare but actionable oncogenic drivers in thyroid carcinoma, particularly in aggressive histologies such as PTDC (10). The role of ALK-targeted therapies is well established in non-small cell lung cancer (NSCLC), but there is no phase II/III evidence about the use of TKI in the thyroid cancer setting.

To date, fewer than ten cases of *ALK*-rearranged thyroid carcinomas treated with ALK TKIs have been reported (**Table 1**). Most involved papillary histology, older patients (ages 64–76), and distant metastases to the lung, brain, or bone. The most frequently reported fusion partner was STRN–ALK, consistent with prior genomic series. First- or second-generation ALK TKIs used include crizotinib, alectinib, ceritinib, and brigatinib. Reported best overall responses (BOR) ranged from partial to complete responses, with time to response typically within the first few months of therapy. However, the durability of response varied, and toxicity, especially gastrointestinal or hepatic, occasionally required dose modifications (12, 16–19).

In this scenario, lorlatinib, a third-generation ALK TKI, has not been studied in *ALK*-rearranged thyroid carcinoma. Lorlatinib has demonstrated significantly longer progression-free survival and better quality of life compared to crizotinib in NSCLC in the CROWN trial and has stronger outcomes than other first and second-generation inhibitors, although there is no direct comparison reported (20).

As illustrated by this case, molecular profiling has become increasingly important in the management of thyroid cancer over the past decade, enabling personalized treatment approaches for advanced disease through the identification of actionable targets and the increasing availability of target therapies (12). Recent European guidelines recommend molecular characterization in patients with thyroid cancer when systemic therapy is being considered, apart from anaplastic thyroid carcinoma, for which genomic testing should be performed at diagnosis. Next-generation sequencing (NGS) is favored for its high sensitivity and specificity; however, its use remains limited by restricted reimbursement and accessibility in thyroid cancer care (21).

Our case is notable for several reasons. This is the only reported patient with PDTC managed with two sequential ALK inhibitors in which the patient achieved a complete metabolic response to lorlatinib after developing resistance to ceritinib, with a sustained response now exceeding 50 months, even after drug discontinuation.

Acquired resistance to second-generation ALK inhibitors is mostly mediated by on-target kinase domain mutations, detected in more than half of cases, with the solvent-front mutation G1202R emerging as the dominant alteration (22). Lorlatinib retains potent activity across the spectrum of single ALK resistance mutations, including G1202R, and has demonstrated clinical efficacy in patients previously treated with second-generation ALK inhibitors, particularly when resistance remains ALK-dependent (23). However, in NSCLC, a subset of tumors progressing on ceritinib lack identifiable ALK resistance mutations and yet remain ALK-dependent (22). In our case, no secondary ALK kinase domain mutation was detected at progression on ceritinib. Persistence of the TFG::ALK fusion without additional oncogenic drivers, together with the profound and durable response to lorlatinib, strongly suggests continued ALK oncogenic dependency rather than activation of a dominant bypass pathway. The greater potency and broader inhibitory profile of lorlatinib may therefore have overcome functional resistance to ceritinib.

Given the scarcity of prospective data, individual case reports remain essential for guiding treatment decisions in this rare molecular subset. This case report shows the efficacy and safety of lorlatinib for the treatment of radioiodine refractory advanced TC. It underscores the importance of assessing *ALK*-rearrangements in this context, demonstrating that clinical benefit from ALK TKIs may extend across histological subtypes and that lorlatinib may provide durable responses after prior ALK TKI failure.

## Data Availability

The original contributions presented in the study are included in the article/**Supplementary Material**. Further inquiries can be directed to the corresponding author.
