# Supplementary figures and images for: Case Report: Lorlatinib for the treatment of ALK-rearranged poorly differentiated thyroid carcinoma after progression to prior ALK-specific tyrosine-kinase inhibitor
Source: Front Oncol. 2026 Mar 9;16:1802225. doi: 10.3389/fonc.2026.1802225 (PMC13006249; doi:10.3389/fonc.2026.1802225)

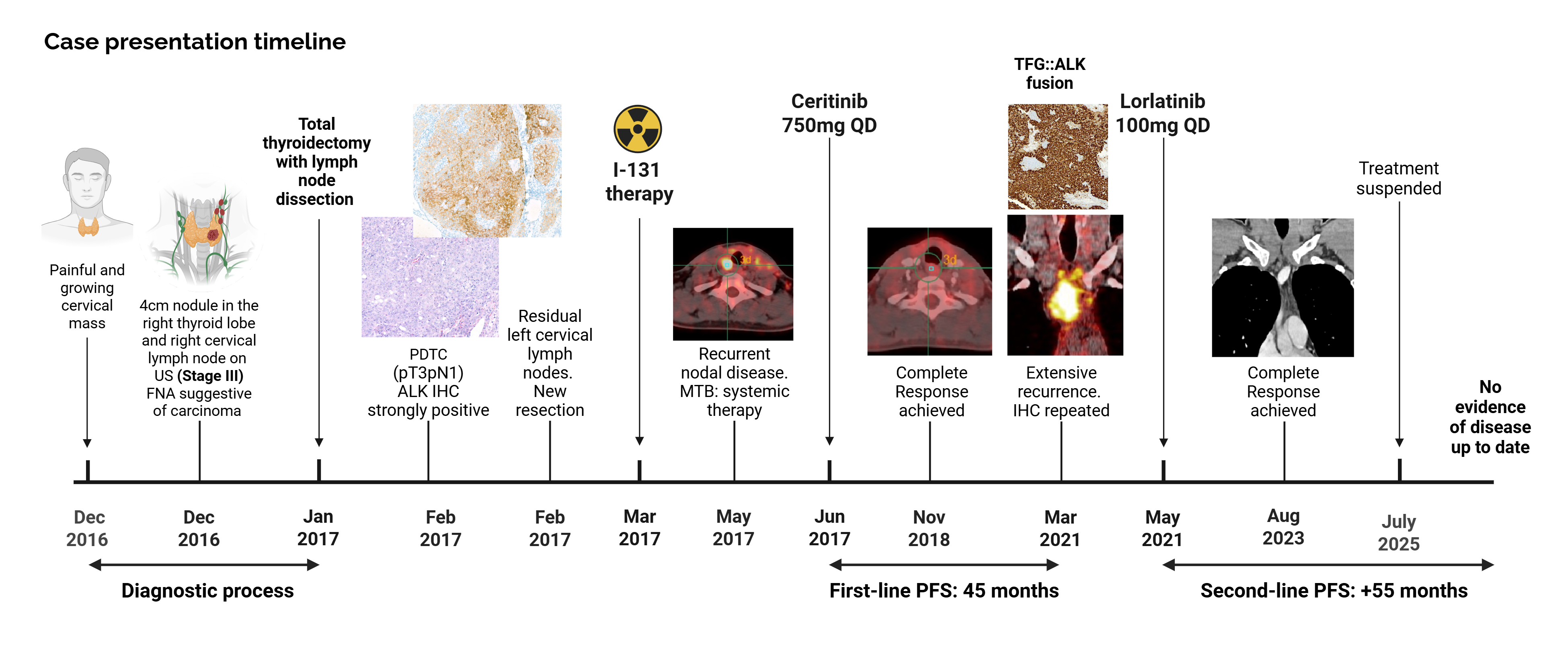

Supplement: Supplementary file 1 [file Image1.jpeg]
